# Bile acids, gut microbiota and metabolic surgery

**DOI:** 10.3389/fendo.2022.929530

**Published:** 2022-08-22

**Authors:** Jui Tu, Yangmeng Wang, Lihua Jin, Wendong Huang

**Affiliations:** ^1^ Department of Diabetes Complications and Metabolism, Arthur Riggs Diabetes and Metabolism Research Institute, Beckman Research Institute, City of Hope National Medical Center, Duarte, CA, United States; ^2^ Irell & Manella Graduate School of Biomedical Science, City of Hope National Medical Center, Duarte, CA, United States

**Keywords:** bile acid, gut microbiota, metabolic surgery, obesity, diabetes, bariatric surgery

## Abstract

Metabolic surgery, or bariatric surgery, is currently the most effective approach for treating obesity and its complications. Vertical sleeve gastrectomy (VSG) and Roux-en-Y gastric bypass (RYGB) are the top two types of commonly performed metabolic surgery now. The precise mechanisms of how the surgeries work are still unclear, therefore much research has been conducted in this area. Gut hormones such as GLP-1 and PYY have been studied extensively in the context of metabolic surgery because they both participate in satiety and glucose homeostasis. Bile acids, whose functions cover intestinal lipid absorption and various aspects of metabolic regulation *via* the action of FXR, TGR5, and other bile acid receptors, have also been actively investigated as potential mediators of metabolic surgery. Additionally, gut microbiota and their metabolites have also been studied because they can affect metabolic health. The current review summarizes and compares the recent scientific progress made on identifying the mechanisms of RYGB and VSG. One of the long-term goals of metabolic/bariatric surgery research is to develop new pharmacotherapeutic options for the treatment of obesity and diabetes. Because obesity is a growing health concern worldwide, there is a dire need in developing novel non-invasive treatment options.

## Introduction

Obesity imposes significant healthcare burden worldwide. The World Health Organization reports that the current number of individuals who have obesity has increased by three-fold since 1975. In 2016, 39% of the adults worldwide were overweight (1). In the United States alone, 20% of the adults had obesity in 2019 (2). These numbers are alarming; according to one report, people who have class III obesity (body mass index, or BMI, ≥40 kg/m^2^) could lose up to almost 14 years in life expectancy (3). There are several comorbidities associated with obesity, such as hypertension, dyslipidemia, cardiovascular diseases, and type 2 diabetes mellitus (T2D) (4, 5). T2D affects many people in the US. The “National Diabetes Statistics Report, 2020” published by the Center for Disease Control and Prevention reports that approximately 34.1 million of adults have diabetes, and T2D accounts for 90-95% of those cases (6).

**Figure 1:**
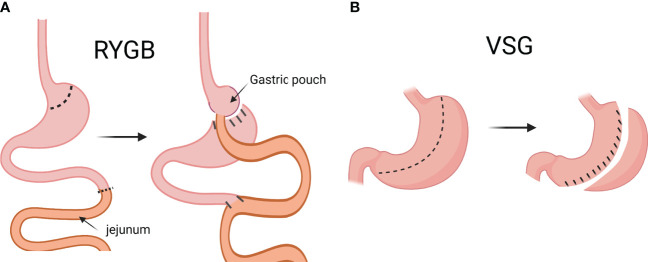
Graphical description of Roux-en-Y gastric bypass (RYGB) and vertical sleeve gastrectomy (VSG). In RYGB, the jejunum is cut, and the distal end is anastomosed to the small gastric pouch, and the proximal end is anastomosed to the rest of the jejunum **(A)**. In VSG, approximately 75-80% of the stomach is removed along the greater curvature to create a sleeve-like gastric pouch **(B)**. (Created with BioRender.com).

Treatments of T2D include lifestyle intervention, pharmacotherapies, and bariatric surgery (7). The term “bariatric surgery” is gradually being replaced by “metabolic surgery” because the surgery is not only recommended for the treatment of obesity, but also other metabolic diseases (8). There are several kinds of metabolic surgery: gastric banding, sleeve gastrectomy (SG; or vertical sleeve gastrectomy, VSG) Roux-en-Y gastric bypass (RYGB), and several others. Right now, VSG and RYGB are the most frequently performed metabolic surgical procedures globally, and the number of VSG performed has been steadily increasing in the US (9). RYGB is the more technically complicated surgery of the two. In brief, the stomach is first divided into two portions: the smaller, proximal pouch, and the larger, distal pouch. Then, the jejunum is cut, and the distal end is anastomosed with the small gastric pouch. The proximal end of the cut jejunum is anastomosed to the rest of the jejunum, distal to the jejunal limb that is anastomosed to the small gastric pouch (**Figure 1A**) (10). The VSG surgery is simpler: approximately 75-80% of the stomach is removed along the greater curvature, leaving a sleeve-like gastric pouch (10, 11). VSG is also often referred to as LSG (laparoscopic sleeve gastrectomy) or simply SG (sleeve gastrectomy). For the sake of consistency, “VSG” will be used throughout the rest of the text, even when referencing publications that originally use a different terminology.

Generally, metabolic surgery is recommended to patients with BMI ≥40 kg/m^2^ (BMI ≥ 37.5 kg/m^2^ for Asian Americans), and who have not successfully achieved adequate weight loss and management of comorbidities (7). While metabolic surgeries are effective, they are not without risks and complications. Intra-operative complications such as bleeding and leakage, and post-operative complications such as hair loss, bone loss, and nutrient deficiency, could all burden patients (11–14). Therefore, there is a clear medical need to identify the underlying mechanisms of action of metabolic surgery, so that new pharmacotherapy options can be developed for treating obesity, T2D, and other metabolic diseases. This review will summarize in the following sections the recent progress made in metabolic surgery research that is related to gut hormones, bile acids and their receptors, and gut microbiota.

## Gut hormones

The feedback loop of hunger, eating, feeling of satiety, and the inhibition of eating behavior is intricately regulated by hormones and peptides (15). Therefore, studying changes in these hormones and peptides after metabolic surgery may provide clues for how the surgery works. Glucagon-like peptide-1 (GLP-1) and peptide tyrosine tyrosine (PYY) are two gastrointestinal hormones that are frequently investigated in metabolic surgery research. GLP-1 is produced by the brainstem and the L cells in the small intestine, and then released upon ingestion of a meal. The release of GLP-1 leads to insulin secretion, reduced hepatic glucose production, reduced food intake, and slowed gastric emptying (16, 17). PYY is also released by the L-cells at the distal small intestine and colon after a meal. Similar to GLP-1, PYY release leads to decreased gastric emptying and suppressed pancreatic secretion (16). In most VSG and RYGB studies, GLP-1 and PYY levels are found to be elevated after the surgeries. In studies done in rats and mice, GLP-1 level was elevated after VSG and RYGB (18, 19). Numerous studies done in humans show similar findings. GLP-1 and PYY are increased after both VSG and RYGB in human patients (20–22), and a systemic review reports that GLP-1 and PYY increased in VSG patients about one year after the surgery (23). A prospective study by Arakawa et al. in human patients also reported that there was a temporal relationship between gut hormone changes and metabolic surgery (24). The authors reported that postprandial GLP-1 level increased in both VSG and RYGB patients at 26 weeks after the surgery. For RYGB patients, their postprandial GLP-1 level was still elevated at 26 weeks after the surgery, and the elevation persisted at 52 weeks after the surgery (24).

Current evidence seems to suggest that gut hormones play important roles in the mechanisms behind metabolic surgery. How VSG and RYGB lead to increase in GLP-1 is believed to be through the alteration in anatomy. GLP-1 production is higher in the distal intestinal tract, and its release is stimulated by carbohydrates, fats, and protein (25). Larraufie et al. found that VSG shortened gastrointestinal transit time of nutrients in mice, and the finding was correlated with an increase in GLP-1 release (26). Further investigation into the roles that gut hormones play in metabolic surgery is needed for finding out how to exploit their therapeutic potential for the treatment of obesity and its comorbidities.

## Bile acids and their receptors

Bile acids are fascinating molecules because they participate in many biological functions. The synthesis of bile acids takes place in the liver, starting with cholesterol. Cholesterol is hydroxylated and modified by several sterol hydroxylases that act on different positions of the cholesterol’s carbon structure. The result is a large variety of bile acid molecules with different degrees of hydrophobicity (27, 28). Traditionally, bile acids are known for their roles in dietary lipid absorption. Upon ingestion of a meal, bile acids are released into the duodenum to begin the process of lipid absorption by emulsifying the lipids (29). When bile acids reach the ileum, they are re-absorbed and circulated back to the liver *via* enterohepatic circulation. The reabsorption of bile acids is very efficient; about 95% of the total bile acid pool is reabsorbed daily, and the rest is excreted in feces and urine (28, 30). Besides lipid absorption, bile acids also function as signaling molecules. Farnesoid X receptor (FXR) and Tekeda-G-protein receptor 5 (TGR5) are two major receptors of bile acids, and their functions will be discussed in a later section (28, 31). Bile acids also interact with gut microbiota; the bi-directional relationship between bile acids and gut microbiota allows them to influence each other’s composition (32). Therefore, bile acids have received substantial interest from the medical and research communities for their therapeutic potential in metabolic diseases.

The roles that bile acids play in metabolic surgery will be discussed in two sections below: Bile Acids, and the Receptors of Bile Acids.

### Bile acids

The composition and kinetics of bile acids have been studied in the context of metabolic surgery in both rodent models and humans. Many studies report that metabolic surgery and its metabolic improvements are associated with the elevation of bile acids in the circulation. Nakatani et al. studied adult obese patients who underwent one of the following metabolic surgeries laparoscopically: RYGB, VSG with duodenal jejunal bypass, VSG, and adjustable gastric banding. The authors found that serum bile acids increased after surgery (33). However, Nakatani et al. did not analyze the surgery types separately. In a later study, Patti et al. focused their study scope on RYGB only, and they also found that total bile acids was significantly higher in individuals who had RYGB than those who were overweight or severely obese (34). The findings in VSG are a bit more varied. A study done in rodents reported that total serum bile acids increased after VSG (35), but a meta-analysis showed that total serum bile acids did not increase in human subjects after VSG (36). In another study by Chen *et al*, the authors reported that after human patients received RYGB and VSG, total bile acids in the blood was increased at both three days and three months after surgery (37).

To better understand the relationship between bile acids and metabolic surgery, it is important to not only look at total serum bile acids level, but also at the alteration of the bile acid composition after metabolic surgery. Ding et al. found that while the total serum bile acids did not change significantly in mice after VSG, the composition of bile acids did: the concentration of taurine-conjugated bile acids increased in the serum after VSG (38). A study done by Wu et al. in a diabetic rat model also reports that besides elevation in total serum bile acids, taurine-conjugated bile acids were elevated after VSG as well (39). One pattern of post-metabolic surgery alteration in bile acid composition that has recently received some attention is the change in the ratio between 12-alpha-hydroxylated (12-α-OH) bile acids and non-12-α-OH bile acids. 12-α-OH and non-12-α-OH bile acids are two major classes of bile acids. In humans, cholic acid (CA), one of the two primary bile acids, is a 12-α-OH BA. The other primary bile acid, chenodeoxycholic acid (CDCA), is a non-12-α-OH bile acids (32). In mice, most members of the non-12-α-OH bile acids are in the form of muricholic acids (MCA) and its associated forms. The ratio between the two classes is determined by the activity of a bile acid synthesis enzyme named sterol-12α-hydroxylase (CYP8B1) because CYP8B1 catalyzes the production of CA (28). High 12-α-OH: non-12-α-OH ratio has been shown to be associated with insulin resistance and obesity in both humans and rodents (40, 41). Rats fed with a Western-style diet were found to produce more 12-α-OH bile acids (42), and mice that were deficient in CYP8B1 were found to be resistant to obesity induced by high-fat diet-feeding due to decreased lipid absorption (43). A recent study performed on a large cohort of VSG patients demonstrated that after the surgery, serum level of CA decreased (a 12-α-OH bile acid), and serum level of taurine-conjugated lithocholic acid (LCA; a non-12-α-OH bile acid) increased (44). Another study reports similar findings: the levels of non-12-α-OH bile acids increased in both RYGB and VSG patients one year after surgery, and the increase was greater in RYGB patients (45). On the contrary, a meta-analysis published by Zhang et al. revealed that after RYGB, the ratio of 12-α-OH: non-12-α-OH bile acids increased instead of decreased in human subjects (36).

Although it is not yet clear why there are differing reports on the post-surgery bile acids composition between RYGB and VSG, what is clear is that the currently available evidence supports the notion that total serum bile acids level and bile acids composition are linked to metabolic surgery. Further research is needed to define how specific bile acids species mediate the health benefits of metabolic surgery. The following section on the functions of bile acids will further underscore the reason for their importance in metabolic surgery research.

### Receptors of bile acids

Bile acids interact with several receptors to regulate physiologic pathways. Different species of bile acids possess different affinity for the receptors. For example, primary bile acids CDCA and CA are potent ligands for FXR, and secondary bile acids like LCA and DCA are potent ligands for TGR5 (46). Therefore, it is crucial to include the receptors in the discussion of how metabolic surgeries work through bile acids.

FXR is a nuclear receptor highly expressed in the liver and the intestine, where bile acids can bind to it directly (47). FXR regulates many genes that are involved in various aspects of metabolism, such as bile acids synthesis and transport, gluconeogenesis, lipogenesis, and fatty acid oxidation, *etc.* (47). Therefore, FXR has been studied extensively in metabolic surgery research. Rodent models are extremely valuable here because they allow genetic modifications to be made, and the collection of tissues for gene and protein expression analysis. Some studies suggest that FXR is required for the success of the surgery. Ryan et al. found that while VSG was successful in bringing significant weight loss to obese WT mice, it failed to do the same in mice deficient of FXR (48). Another group investigated the role of FXR in RYGB surgery: Kong et al. performed RYGB on spontaneous diabetic Goto-Kakizaki rats, and found that CDCA, a potent agonist of FXR, was increased in serum significantly after RYGB (49). The capacity of pancreatic β-cells to secrete insulin also increased after RYGB. However, when RYGB was performed in FXR-deficient mice, their pancreatic β-cells did not improve in insulin secretion (49). On the other hand, some publications report that FXR is not required for metabolic surgery to bring forth metabolic improvement. Li et al. showed that RYGB induced loss of body weight in both WT and FXR-deficient mice (50), even though FXR-deficient mice did not improve in glycemic control following RYGB the same way that WT mice did. By using mice that were deficient in FXR specifically in the liver and the intestine, Ding et al. reported that VSG was still able to improve metabolic parameters in mice (51). The study also shows that perhaps instead of FXR, the decreased intestinal bile acid level and subsequently decreased lipid absorption are part of the underlying mechanism of metabolic surgery.

The variability in the reports of how FXR plays a role in metabolic surgery is not surprising, considering the wide range of its tissue expression and physiologic processes that it mediates. Therefore, analysis of FXR’s downstream targets may be a good direction for finding the underlying mechanisms of metabolic surgery (52). One such target is the gut-derived hormone fibroblast growth factor (FGF) 15 or 19 (FGF15 in mice, and FGF19 in humans). FGF15/19 is produced by the enterocytes in the ileum, and it is released after FXR activation. Once released, FGF15/19 then enters the circulation to reach the liver, where it can bind to its receptor FGFR4. Finally, FGF15/19 completes the negative feedback loop of bile acids synthesis by suppressing the rate-limiting enzyme of bile acids synthesis in the liver, CYP7A1 (28, 46, 52). The significance of FGF15/19 in metabolic surgery has been investigated in both animal and human studies. In FGF15-deficient mice, VSG caused significant weight loss but did not improve glucose tolerance (35). In human patients that received VSG or RYGB, Chen et al. found that FGF19 levels increased at three days following both surgeries. However, by three months after the surgeries, the levels were no longer different between the groups (37). In another study that followed up with patients one year after VSG and RYGB, Nemati et al. reported that FGF19 increased after both VSG and RYGB, and the level of increase was similar between the two groups (45). Additionally, the increase of FGF19 was found to be correlated with T2D improvement. Available evidence suggests that it is worthwhile to investigate FGF15/19 further as a potential player behind metabolic surgery.

Besides FXR, another receptor that bile acids interact with is TGR5. Unlike FXR, TGR5 is a membrane-bound G protein-coupled receptor. Activation of TGR5 leads to the stimulation of adenylate cyclase, production of cAMP, then finally activation of protein kinase A. These processes lead to the modulation of various inflammation and metabolism functions, such as bile acids homeostasis, GLP-1 production, insulin sensitivity, and energy expenditure (47). In contrast to FXR, TGR5 has stronger affinity for secondary bile acids (LCA more than DCA) than primary bile acids. Taurine-conjugated bile acids also produce higher potency at TGR5 than unconjugated and glycine-conjugated bile acids (46). TGR5 mediates the outcome of metabolic surgery in the aspects of glucose regulation and bile acid composition. Mice that were deficient in TGR5 showed dampened response to VSG in their metabolic improvements compared to the WT control mice (38). Moreover, McGavigan et al. found that the shift in bile acids composition that is usually observed after VSG surgery, namely, the decrease in 12-α-OH/non-12-α-OH bile acids ratio in serum, was not observed in TGR5-deficient mice after VSG surgery (53). On the contrary, a study conducted in mice reported that TGR5 is not necessary for the health benefits of RYGB (54).

The evidence mentioned in this section not only reinforces the notion that bile acids composition is an important mediator of the beneficial changes that metabolic surgery brings, but also provides insights into how bile acids and associated molecular targets may be part of the equation of how metabolic surgery works.

## Gut microbiota

**Figure 2:**
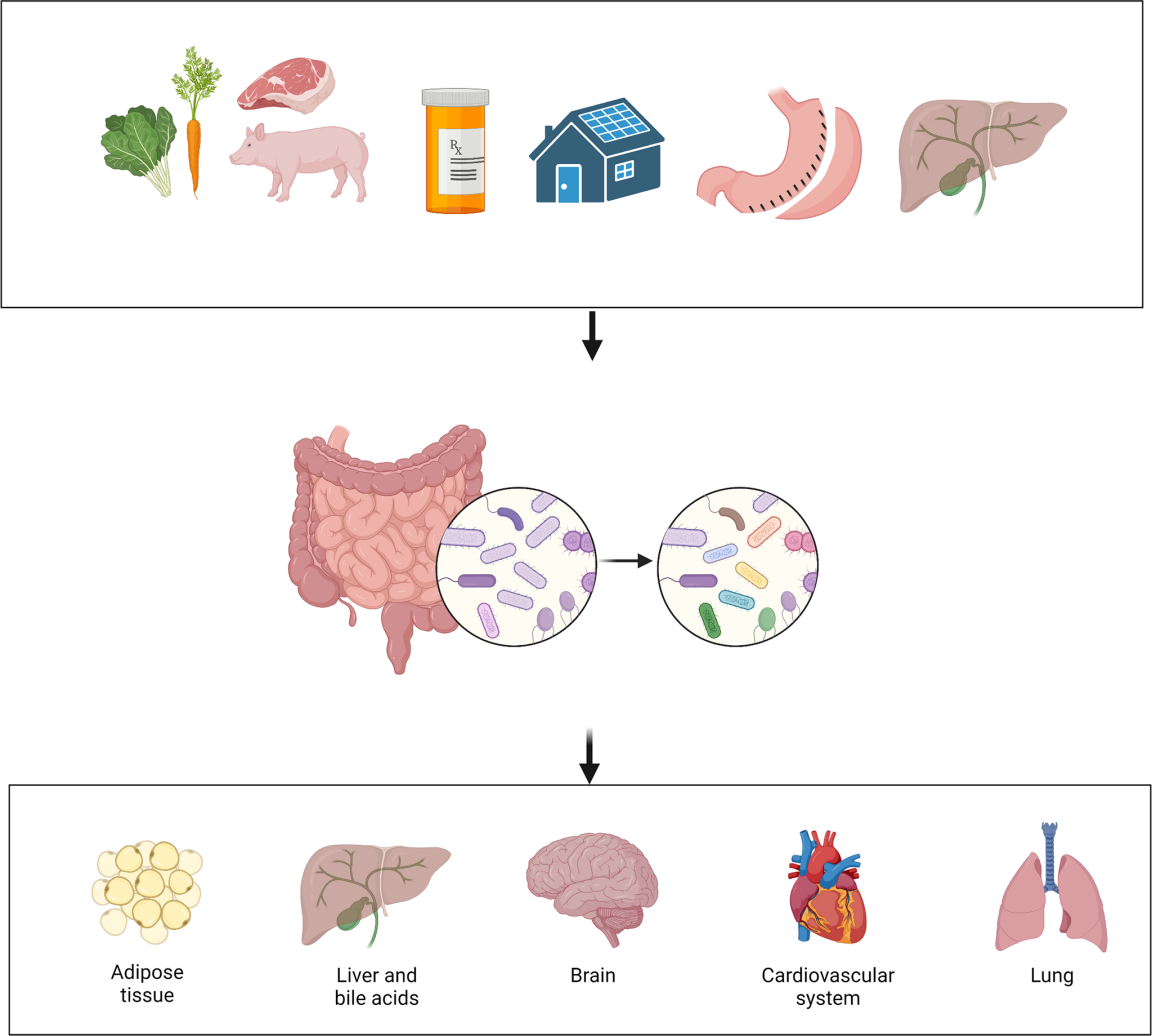
Interaction among environment, gut microbiota and host. Many factors can affect the gut microbiota, such as diet, medication, environment, bariatric surgery, and bile acids. Alteration in the gut microbiota can then affect the health status of multiple organ systems. (Created with BioRender.com).

The microbial communities that reside in an individual person or animal are numerous and diverse. The estimated number of microbes that inhabits the colon of an adult human is 3.2 x 10^11^ cells per gram of content (55). Growing evidence shows that the gut microbiota is involved in a large variety of physiologic and pathologic processes. Many factors can affect the composition of gut microbiota, such as diet, medication, and external environment (**Figure 2**) (56). “Normal” or “healthy” composition of gut microbiota contributes to physiologic processes such as nutrient extraction and the development of immune system (57). Disruption of the normal composition could lead to alteration in the health status of organ systems such as the brain, the heart, and the lung (56, 58, 59). Specifically, mental health status (60), inflammatory responses (61, 62), and even pain perception (63, 64), can all be affected by the gut microbiota.

Gene sequencing technologies such as 16s rRNA sequencing and shotgun metagenomic sequencing, combined with powerful analytic tools, allow for the extraction of genetic and functional information from samples (65). Most of the gut bacteria in humans and laboratory rodents belongs to two major phyla: Firmicutes and Bacteroidetes (57, 66). Studies found that the relative abundance of the two phyla is associated with obesity. In both mice and humans, obesity is reported to be associated with higher ratio of Firmicutes: Bacteroidetes (66–68). However, some studies found either opposite or lack of association between obesity status and Firmicutes: Bacteroidetes ratio (69, 70). This conflict could be the result of technical differences between studies, or due to the complex nature of obesity and microbiota (70).

As the field of gut microbiota research progresses, more and more studies report gut microbiota data that are beyond the phylum level. Studies conducted in mice show that some species are associated with metabolic disturbance. For example, several species of *Lactobacillus* have been reported to have the ability to prevent weight gain and blood glucose disorder in mice that were fed high-fat diet (71). The results from a clinical trial supported the beneficial effect of *Lactobacillus* spp, showing that overweight subjects who consumed yogurt containing heat-killed *Lactobacillus plantarum* OLL1712 displayed significantly less abdominal fat accumulation and lower fasting plasma glucose (72). Similarly, *Akkermansia muciniphila* and *Parabacteroides distasonis* have both been deemed beneficial to metabolic health (73–75).

Gut microbiota has also been investigated as part of the underlying mechanism of metabolic surgery. The close interaction between gut microbiota and bile acids makes studying the gut microbiota in this context particularly interesting. The following sections will introduce the roles that gut microbiota may play in metabolic surgery. First, the interaction between the gut microbiota and bile acids will be introduced. Then, the way that the gut microbiota is influenced by metabolic surgery will be discussed, and the discussion will also include changes in adipose tissues after metabolic surgery. Finally, the discussion of gut microbiota will be concluded with how certain metabolites of gut microbiota could be exploited as therapeutic options for obesity, T2D, and other metabolic diseases.

### Gut microbiota and bile acids

The gut microbiota possesses the ability to modulate the composition of bile acids. The production of secondary bile acids relies on the hydroxylation and dehydroxylation carried out by the gut microbiota at the distal small intestine and the colon (32, 76). Bile acids are conjugated mostly with glycine in humans, and with taurine in rodents, which increases their solubility (32, 77). The gut bacterial species that have bile salt hydrolase can deconjugate bile acids from taurine and glycine; then, further modification by other mechanisms of the gut bacteria results in the production of secondary and tertiary bile acids (77). The gut microbiota can also influence the activity of FXR and TGR5 through altering the composition of bile acid pool (32). The impressive impact that gut microbiota has on bile acid composition and the genes that regulate bile acid synthesis is perhaps best demonstrated in germ-free mice (GF). Compared to conventionally-raised mice, GF mice showed a lack of secondary bile acids, decreased overall bile acid pool size, and altered composition of bile acids at various segments of the intestines (78). Additionally, the expression levels of bile acid synthesis enzymes CYP7A1, CYP7B1, CYP8B1, and CYP27A1 have also been found to be different between GF and conventionally-raised mice (78, 79). These findings further support the notion that gut microbiota can impact bile acid composition.

Bile acids can influence the composition of gut microbiota as well. Bile acids have long been known to have antimicrobial property. An *in vitro* study showcased the antimicrobial activity of bile acids against *Staphylococcus aureus* (80) by demonstrating that CA and DCA decreased the viability of *S. aureus* in a concentration-dependent manner. In mice, feeding of ursodeoxycholic acid (UDCA) altered both the microbiota and bile acid compositions (81). In rats, feeding of CA for 10 days increased the proportion of the Firmicutes phylum in the gut microbiota (82).

The relationship between bile acids and gut microbiota is bi-directional. It should be no surprise, then, that the gut microbiota has received much attention in the realm of metabolic surgery research.

### Gut microbiota and metabolic surgery

The implication of gut microbiota in how metabolic surgery works has been acknowledged for some time now. Many studies have reported on the shifts in gut microbiota in rodents and humans after metabolic surgery. Taken into consideration of the bi-directional relationship between bile acid and gut microbiota, how metabolic surgery influences the gut microbiota (or vice versa) could hold the key to uncovering the underlying mechanisms of metabolic surgery.

The early investigation of the role of gut microbiota in metabolic surgery was focused on finding trends or patterns of how gut microbiota changed after metabolic surgery. In a small study of nine human subjects, changes in fecal microbiota were detected between individuals that were lean, morbidly obese, and after RYGB (83). Phylogenic analysis revealed that the microbiota communities tended to cluster together in individuals within the same cohort, with pronounced distinction between lean and obese individuals. A later study investigated the alteration in fecal microbiota after dietary intervention aimed at treating obesity, and after VSG (84). The authors found that although similar degree of weight loss was achieved by both groups, the ways that the microbiota compositions altered were not the same. After dietary intervention, the proportion of Bacteroidetes phylum decreased, and the proportion of Firmicutes phylum increased; but the opposite changes were observed after VSG. Similarly, a recent study that also analyzed the gut microbiota of individuals who received dietary interventions or VSG reported no common pattern of microbiota changes between the groups (85). These reports showed that although gut microbiota can be influenced by the metabolic health status of an individual, it can also be influenced by the type of intervention the individual receives. In other words, it is possible that metabolic surgery places a unique signature on the gut microbiota.

Several studies also compare how different types of metabolic surgery could alter the gut microbiota. Gastric banding surgery does not require drastic anatomic alteration like VSG and RYGB do, so it is not surprising that the gut microbiota was not significantly affected in human subjects after gastric banding surgery (86). However, the same study also found that the gut microbiota of the human subjects who received RYGB was significantly different from subjects who did not receive the surgery. A more recent study compared VSG and RYGB surgeries in human subjects, and the authors reported that VSG imposed more prominent effect on gut microbiota than RYGB (87). The authors found that after VSG, 23 bacterial genera increased in abundance and 10 genera decreased; after RYGB, 19 genera increased in abundance and one decreased. It is important to note that among the differences, there are also similarities; of the affected genera, VSG and RYGB shared 10 of the increased genera, and one of the decreased genera.

The reason why dietary intervention and different types of metabolic surgeries alter the gut microbiota differently is still being investigated. If the gut microbiota contains the ability to influence metabolism, then fecal microbiota transplantation (FMT) experiments may help answer some questions. Liou et al. performed FMT experiment in which feces from mice that received RYGB or sham surgery were transplanted to GF mice. The results showed that the body weight and adiposity of the recipients of RYGB feces were lower than the recipients of sham feces (88). Later, Groot et al. conducted a FMT study with human subjects: fecal microbiota from human subjects who had metabolic syndrome and who received RYGB surgery were transferred to nonsurgical subjects with metabolic syndrome (89). The results showed that while recipients of gut microbiota from donors with metabolic syndrome had worsened insulin sensitivity, recipients of gut microbiota from RYGB donors showed trends of improvement in insulin sensitivity, although the improvement was statistically insignificant. There is clearly much more to discover and investigate in the role that gut microbiota plays in the beneficial effects of metabolic surgery.

Efforts have been made to identify bacterial species that can mediate the health benefits of metabolic surgery. One candidate is *Akkermancia muciniphila.* Abundance of *A. muciniphila* was found to be lower in leptin-deficient obese mice and high-fat-diet-fed mice than in lean mice (90). When the bacterium was administered to the mice, body weight and body composition improved. Similar findings have been reported in humans. A proof-of-concept study published by Depommier et al. shows that *A. muciniphila* could be safely administered to human volunteers. Although the changes were not significant, the authors found that *A. muciniphila* treatment had beneficial metabolic effects such as improvement in insulin sensitivity, reduction of insulinemia, and loss of body weight (75). However, there are conflicting reports. A recent study looked at the abundance of *A. muciniphila* in patients after gastric banding and RYGB surgeries (91). *A. muciniphila* was not increased in gastric banding patients, but it was increased in RYGB patients. The authors also reported that the abundance of the bacterium at baseline was not correlated with clinical outcome after RYGB, and after RYGB the increase in the abundance of *A. muciniphila* was not correlated with glucose homeostasis and other clinical variables. The question of whether or not *A. muciniphila* or any one bacterium has the ability to effectively treat obesity is still being studied. Instead of focusing on the bacteria themselves, some groups have turned their attention to the metabolites of the bacteria. In the next section, how the metabolites and functions of the gut microbiota can be exploited for metabolic health benefits will be discussed.

### Metabolites and functions of gut microbiota, and their therapeutic values

Metabolomics is another area of focus in metabolic surgery research. Metabolomic studies may enhance the efforts of mining gut microbiota for mechanistic clues by narrowing down the physiologic pathways that are impacted after metabolic surgery. Then, the alteration in gut microbiota composition can be taken into account while studying the impacted pathways. Because identifying specific bacterial species or groups thar bear therapeutic potentials for obesity is challenging, redirecting our attention to the physiologic pathways and metabolites over which the surgery-associated gut microbiota profile has influence may be a more practical strategy.

A tryptophan-derived metabolite named indole-3-acetic acid (IAA) has been studied for its association in metabolic health. IAA levels in the serum was lower in HFD-fed mice, and correspondingly, the abundance of the gut bacteria that metabolize tryptophan to produce IAA also was found to be decreased (92). One of the consequences of obesity is non-alcoholic fatty liver disease (NAFLD) (93), and Yu et al. explored the role of IAA in improving NAFLD after VSG (94). The authors found that in human patients, NAFLD was improved after VSG, and serum IAA level was increased at both one and three months after the surgery. By using mice, the authors established the link between IAA and NAFLD by administering IAA to HFD-fed mice. As expected, administration of IAA improved NAFLD and increased the number of anti-inflammatory macrophages in the livers of HFD-fed mice. Some studies looked at bacterial functional pathways at various time points after metabolic surgery. Shen *et al*. reported that 15 bacterial functional pathways were enriched in post-RYGB patients compared to before surgery (95). Examples of these pathways are metabolism of amino acids, carbohydrates, lipids, and vitamins, etc. However, 12 of these pathways regressed to pre-surgery levels at 12 months after RYGB, despite sustained weight loss. Analysis of the alteration in gut microbiota at pre-surgery and 12 months after surgery revealed similar regression. The question of how much gut microbiota can influence metabolic surgery outcome is still up for debate. Shen *et al’s* results show that it is possible for the surgery to overpower the influence of gut microbiota.

### Metabolites and the effects of metabolic surgery on adipose tissues

Metabolic surgeries efficiently reduce body mass and adiposity. Adipose tissues are remodeled after VSG with smaller fat pad and adipocyte size. Growing evidence showed that VSG induced microbiota and metabolites alteration have key effects on reduced fat mass. As an endocrine organ, gut microbiota produced metabolites like bile acids, SCFA (short chain fatty acids) and BCAA (Branched-Chain Amino Acids) have been reported to regulate lipid metabolisms in adipose tissue. These metabolites have potential regulatory roles in metabolic surgery induced fat loss.

#### Bile acids

The level and composition of bile acids are known to be altered by metabolic surgery. Our previous study showed that after VSG surgery, remodeled bile acids activate TGR5-cAMP signaling pathway in brown adipose tissue (BAT) and promote BAT thermogenesis. TGR5^-/-^ mice failed to maintain VSG-induced body weight loss, BAT activity and energy expenditure (38). It has also been reported that bile acid–TGR5 axis promotes white fat browning and lipolysis (96). VSG induced elevation of conjugated bile acids have more potency to activate TGR5 than un-conjugated bile acids (97). Bile acid-TGR5 signaling plays a key role in reduced adiposity after VSG (38). Interestingly, compared with bariatric surgery, microbiota and bile acids alteration after caloric restriction (CR) are responsible for rebound weight gain in mice. CR caused dramatically increased proportion of non-12α-OH bile acids, ursodeoxycholic acid and lithocholic acid. These alterations lead to decreased UCP1 expression in brown adipose tissue of weight rebounded mice (98). The difference of bile acids level and composition between bariatric surgery and CR explains why bariatric surgeries are more effective in maintenance of lower body weight than CR.

#### BCAA

BCAAs, including leucine, isoleucine, and valine are essential amino acids which can be synthesized or degraded by gut bacteria. Obesity increases, while bariatric surgery decreases the circulating levels of BCAAs. Mice fed with BCAA deficient diets exhibit reduced body weight and adiposity, accompanied with reduced lipogenesis and increased lipolysis in white adipose tissue. (99–101). But another publication showed that decreased circulating BCAAs is not required for VSG induced weight loss (102). When mice fed with HFD supplemented with BCAAs were subjected to VSG surgery, sustained weight loss and improved glucose tolerance were identical to mice fed with regular HFD. Impaired BCAA catabolism by depletion of Pp2cm didn’t affect VSG induced weight loss. This study suggests that although circulating BCAAs level is reduced after VSG, it’s not the driver of VSG induced weight loss.

#### SCFA

SCFA produced by anaerobic intestinal microbiota has been known to be involved in the regulation of immune response and glucose and lipid metabolism. A previous study showed SCFA acetate plays an important role in regulating human adipose tissue lipolysis. Acetate can reduce phosphor-HSL level and lipolysis in human white adipocyte (103). After metabolic surgery, total level of fecal SCFAs was reduced. Among the SCFAs, acetate, propionate, and butyrate were reduced, while the branched SCFAs isobutyrate, isovalerate and isocaproic acid were increased (104). However, the effects of SCFAs on metabolic surgery induced fat mass loss still need to be elucidated.

Many other bacterial metabolites have been investigated, such as lipopolysaccharides, aromatic amino acids, and methylamines (105). More research is needed to discover the connection between bacterial metabolites and metabolic surgery. The complexity of the subject highlights the need for unbiased reporting of both positive and negative results, so that the scientific community can take advantage of all the available knowledge and take the next steps towards developing new therapies for obesity and metabolic diseases.

## Adipocyte-derived exosomal miRNA

Exosomes are nanosized extracellular lipid bilayer vesicles secreted from cells which contain nucleic acids, proteins and lipids. By transferring the biological information to other cells or tissues, exosomes play key roles in intracellular communication and biological activities. Exosomes derived from adipose tissue have been linked to insulin resistance in obese individuals (106). Growing evidence indicates that adipocyte-derived exosomal miRNAs target adipose tissue and distal organs, primarily liver, to regulate metabolic gene expressions (107). Recent studies showed that after bariatric surgery, circulating exosomal miRNA derived from adipocyte significantly changed which correlated to improvements in insulin sensitivity (106). Alteration of adipocyte-derived exosomal miRNA after bariatric surgery provides a novel way to understand the underlying mechanism of the metabolic improvements caused by bariatric surgeries.

## Perspective

Identifying the underlying mechanisms of metabolic surgery is an enormous endeavor. There are likely multiple mechanisms, all of them interconnected in some ways. There is still much to learn about the physiological changes after metabolic surgery. knowledge gained from studying the post-surgery changes could provide clues to how the surgery works. Gut hormones, bile acids, and gut microbiota are just some of players that are investigated. The gut microbiota influences many aspects of metabolism, but the extent to which it can influence the outcome of metabolic surgery is still being investigated. The metabolites of gut microbiota have been receiving more attention, and they may be harboring important clues for developing new therapeutics for treating obesity.

**Figure 3:**
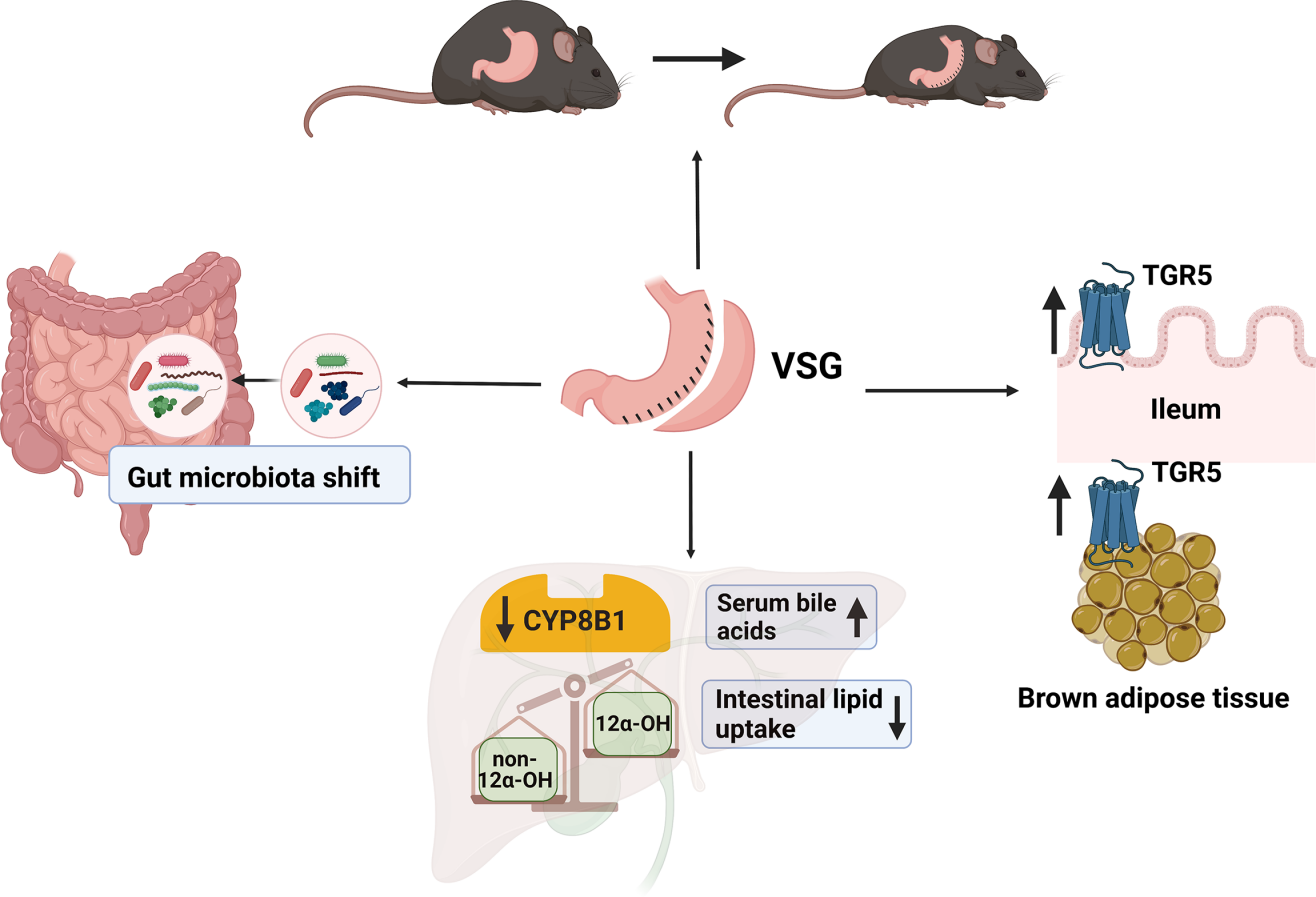
A summary of what we know about VSG, bile acids, and gut microbiota. After VSG in mice, bile acid receptor TGR5 in the ileum and brown adipose tissue is activated, and subsequently leads to increased energy expenditure and decrease in body weight (109). Total serum bile acids is increased after VSG, and intestinal lipid uptake is decreased. The downregulation of CYP8B1 after VSG leads to a decrease the ratio of 12α-OH and non-12α-OH bile acids. Finally, the gut microbiota profile is shifted after VSG; but the precise relationship between VSG and gut microbiota needs further investigation. (Created with BioRender.com).

Readers may refer to **Table 1** for a summary of the key references mentioned in this text, and their main findings. **Figure 3** is a graphical summary of what is known about VSG, bile acids and the gut microbiota.

**Table 1:** List of potential mechanisms underlying metabolic surgery, and how they are affected by the surgery.

Mechanism	Surgery type	Study subject	Effect	Reference
GLP-1	RYGB and VSG	Animal (Rat)	Increased	(18)
	VSG with transit bipartition			
	VSG	Animal (Mouse)	Increased	(19)
	VSG and RYGB	Human	Increased	(24)
GLP-1 and PYY	VSG and RYGB	Human	Increased	(20, 21)
	VSG	Human	Increased	(23)
		Animal (Mouse)	Increased	(26)
PYY	VSG and RYGB	Human	Increased	(22)
Bile acids	VSG, VSG with duodenal-jejunal bypass, RYGB, and adjustable gastric banding	Human	Increased: total serum bile acids.	(33)
	VSG and duodenal-jejunal bypass	Animal (Rat)	Increased: total serum bile acids. Increased: Taurine-conjugated bile acids.	(39)
	VSG and RYGB	Human	Increased: serum secondary and conjugated bile acids.	(37)
			Increased: non- 12α-OH bile acids.	(45)
	RYGB	Human	Increased: 12α-OH bile acids.	(36)
			Increased: total serum bile acids.	(34)
	VSG	Animal (Mouse)	Increased: total serum bile acids.	(35)
			No significant difference: total serum bile acids. Increased: serum concentration of unconjugated and taurine-conjugated bile acids.	(38)
		Human	Increased: LCA in the serum. Decreased: conjugated and unconjugated CA in the serum.	(44)
FXR	RYGB	Animal (Rat)	CDCA, a potent ligand for FXR, was elevated after RYGB. Pancreatic β-cells from FXR-deficient mice did not improve in insulin secretion after RYGB.	(49)
		Animal (Mouse)	FXR was not required for RYGB to induce metabolic changes in mice.	(50)
	VSG	Animal (Mouse)	FXR-deficient mice did not benefit from VSG	(48)
			Liver- and intestine-FXR tissue specific knockout mice still responded to VSG.	(51)
FGF15	VSG	Animal (Mouse)	FGF15-deficient mice lost weight but did not improve glucose tolerance after VSG	(35)
FGF19	VSG and RYGB	Human	Increased at 3 days after surgeries, but decreased back to baseline at 3 months after surgeries	(37)
			Increased at 1 year after VSG and RYGB.	(45)
TGR5	RYGB	Animal (Mouse)	Mice deficient in TGR5 still benefited from RYGB.	(54)
	VSG	Animal (Mouse)	Increased; mice deficient in TGR5 showed dampened response to VSG.	(38)
			Mice deficient in TGR5 showed dampened response to VSG. Mice deficient in TGR5 did not show decrease in the ratio of 12-α-OH and non-12-α-OH bile acids after VSG.	(53)
TGR5 and bile acids	VSG	Animal (Mouse)	Mice had increased amount of CA7S (sulfated CA) after VSG, and CA7S acted on TGR5 to induce anti-diabetic effects.	(108)
Gut microbiota	RYGB	Human	Firmicutes phylum decreased after RYGB.	(83)
	VSG and dietary intervention	Human	After VSG, patients had increased abundance of Bacteroidetes and decreased abundance of Firmicutes. After dietary intervention, patients had decreased abundance of Bacteroidetes and increased abundance of Firmicutes.	(84)
			Gut microbiota pattern is more associated with the particular type of weight loss intervention than weight loss alone.	(85)
	Gastric banding and RYGB	Human	RYGB altered gut microbiota to a greater degree than gastric banding did.	(86)
			The abundance of *A. muciniphila* was not increased in patients after gastric banding, but it increased after RYGB. The increase in *A. muciniphila* was not correlated with clinical variables of metabolic health.	(91)
	VSG and RYGB	Human	VSG imposed greater alteration on gut microbiota than RYGB did.	(87)
	RYGB	Animal (Mouse)	FMT: after receiving feces from post-RYGB mice, recipient mice showed reduced body weight and adiposity.	(88)
		Human	FMT: feces from RYGB patients were giving to non-surgical obese recipients, and the recipients showed improved insulin sensitivity (though statistically insignificant).	(89)
Gut microbiota		Human	Bacterial functional pathways were modified after RYGB, and most modifications regressed at 12 months after surgery.	(95)
Gut microbiota metabolites	VSG	Human and animal (Mouse)	IAA was increased in the serum of patients after VSG. IAA administration to mice improved NAFLD.	(94)

## Author contributions

WH and JT prepared the manuscript. YW and LJ helped edit and provide related information. All authors contributed to the article and approved the submitted version.

## Funding

This study was supported by grants from the George & Irina Schaeffer Foundation, the John Hench Foundation, and the National Institutes of Health (R01DK124627) to WH. WH was also supported by the National Institutes of Health (R01CA139158).

## Conflict of interest

The authors declare that the research was conducted in the absence of any commercial or financial relationships that could be construed as a potential conflict of interest.

## Publisher’s note

All claims expressed in this article are solely those of the authors and do not necessarily represent those of their affiliated organizations, or those of the publisher, the editors and the reviewers. Any product that may be evaluated in this article, or claim that may be made by its manufacturer, is not guaranteed or endorsed by the publisher.
